# Complex Hippocampal Response to Thermal Skin Injury and Protocols with Hyperbaric Oxygen Therapy and *Filipendula ulmaria* Extract in Rats

**DOI:** 10.3390/ijms25053033

**Published:** 2024-03-06

**Authors:** Bojana Krstic, Dragica Selakovic, Nemanja Jovicic, Milos Krstic, Jelena S. Katanic Stankovic, Sara Rosic, Dragan Milovanovic, Gvozden Rosic

**Affiliations:** 1 Department of Physiology, Faculty of Medical Sciences, University of Kragujevac, 34000 Kragujevac, Serbia; b.barlov_92@hotmail.com (B.K.); krsticmilos@hotmail.rs (M.K.); grosic@fmn.kg.ac.rs (G.R.); 2 Department of Histology and Embryology, Faculty of Medical Sciences, University of Kragujevac, 34000 Kragujevac, Serbia; nemanjajovicic.kg@gmail.com; 3 Department of Science, Institute for Information Technologies Kragujevac, University of Kragujevac, 34000 Kragujevac, Serbia; jkatanic@kg.ac.rs; 4 Clinical Pharmacology Department, Clinical Centre Kragujevac, 34000 Kragujevac, Serbia; piki@fmn.kg.ac.rs; 5 Department of Pharmacology and Toxicology, Faculty of Medical Sciences, University of Kragujevac, 34000 Kragujevac, Serbia

**Keywords:** hippocampus, thermal skin injury, anxiety, hyperbaric oxygen therapy, *Filipendula ulmaria* extract, rats

## Abstract

The aim of this study was to evaluate the alterations of the hippocampal function that may be related to anxiogenic response to thermal skin injury, including the morpho-functional alterations, and the effects of hyperbaric oxygen (HBO) and *Filipendula ulmaria* (FU) extract in the treatment of anxiety-like behavior that coincides with thermal skin injury. A rat thermal skin injury experimental model was performed on 2-month-old male *Wistar albino* rats. The evaluated therapeutic protocols included HBO and/or antioxidant supplementation. HBO was applied for 7 days in the hyperbaric chamber (100% O_2_, 2.5 ATA, 60 min). Oral administration of FU extract (final concentration of 100 mg/kg b.w.) to achieve antioxidant supplementation was also applied for 7 days. Anxiety level was estimated in the open field and elevated plus-maze test, which was followed by anesthesia, sacrifice, and collection of hippocampal tissue samples. HBO treatment and FU supplementation significantly abolished anxiogenic response to thermal skin injury. This beneficial effect was accompanied by the reduction in hippocampal pro-inflammatory and pro-apoptotic indicators, and enhanced BDNF and GABA-ARα2S gene expression, previously observed in untreated burns. The hippocampal relative gene expression of melatonin receptors and NPY positively responded to the applied protocols, in the same manner as µ and δ opioid receptors, while the opposite response was observed for κ receptors. The results of this study provide some confirmations that adjuvant strategies, such as HBO and antioxidant supplementation, may be simultaneously applied in the treatment of the anxiety-like behavior that coincides with thermal skin injury.

## 1. Introduction

Thermal skin injuries are the most common type of burns, making up about 86% of burned patients requiring burn center admission. They are caused by excessive heat, hot liquids (scalds), hot solids (contact burns), flames (flame burns), and electrical injury [1]. Burns are physically and psychologically challenging injuries, with a high incidence of disability and mortality. Extensive burns require long-term treatment in specialized centers and therefore represent a major health economic and social problem [2].

In addition to local changes caused by the direct effect of the thermal agent, burn injury is also accompanied by a systemic response of the organism, which can potentially result in multiple organ dysfunction syndrome. Prognosis and treatment outcome directly depend on the surface and depth of the burn, as well as on the patient’s age, health status, and accompanying complications [3]. Major complications include shock, wound infection, pulmonary infection and injury, acute renal failure, neurological sequela, and multi-organ failure [4].

Previous scientific data indicate that central nervous system (CNS) morbidity is significantly increased after burn trauma and a considerable number of burned patients with any severity showed neurological disorders, such as persistent headache, memory loss, and paresthesia [5]. Cognitive dysfunctions, such as memory impairment, are major neurological sequelae affecting the quality of life of burn patients [6]. The condition of acute neurological dysfunction caused by thermal trauma can be referred to as burn encephalopathy. Most burn patients are disturbed by anxiety, depression, and insomnia [7].

Destruction of tissue by direct effect of a thermal agent, as well as subsequent surgical interventions and physical rehabilitation, induces pain that is recognized as one of the main causes of anxiety in burn patients [8]. Damage or loss of functioning in burn survivors, changes in physical appearance due to scarring, long absences from loved ones due to long hospitalizations, and physical rehabilitation lead to thoughts of discomfort, worry, and fear in patients, and common challenges of everyday life can present psychological stress [9]. It has been observed that most patients with superficial burns suffer from mild anxiety and patients with deep burns may suffer from severe anxiety, while the association between facial burns and the severity of anxiety is very high, indicating that facial deformity is a risk factor for burns anxiety [10,11]. Alvi and colleagues reported that of 100 burn patients who were hospitalized, 82% had anxiety, so treating these disorders could improve the quality of the patients’ experience and ensure better outcomes [12].

The various clinical challenges in treating burn patients include dealing with many factors that affect the length of hospital stay, cost of treatment, risk of infection, time to wound healing, length of rehabilitation, and successful reintegration of survivors into society with a healthy mind and body [13]. Over the past few decades, great advances have been made in the treatment and care of burn patients, resulting in increased survival, shorter hospital stays, and lower treatment costs. These advances in treatment have allowed researchers to pay attention to other effects of burn trauma, such as the psychological ones [4]. An initial focus on physical limitation in burn treatment is necessary, but it must also address psychological trauma, which may last longer than physical limitation. Therefore, burn treatment is a multidisciplinary approach, as the focus is on restoring functionality both physically and mentally [14].

Hyperbaric oxygen (HBO) therapy, which is based on the use of 100% oxygen at pressure greater than one atmosphere, belongs to the auxiliary treatments in the treatment of burns, due to its therapeutic properties of reducing tissue hypoxia, pathological inflammation, and stimulating neovascularization. There is a lot of experimental and clinical research on the positive effect of HBO therapy on burn healing. The results of clinical trials have not so far led to full agreement about the benefits of HBO application to the reduction in the length of stay in hospital [15], and consequently, to the total treatment cost. Having in mind that burns can cause significant, painful wounds that alter normal protective physiology of the skin and thus affect psychological well-being of burn patients, Rasmussen and coworkers conducted a study evaluating the antinociceptive effect of hyperbaric oxygen therapy after a thermal injury. It has been shown that HBO therapy in humans reduces central sensitization caused by thermal skin injuries and thus lowers the thermal pain threshold [16]. Also, a recent study in rats with third-degree burns showed that prolonged administration of HBO therapy can reduce burn-induced mechanical allodynia for a longer period of time in rats [17].

High temperatures during burns break the chemical bonds between skin molecules, leading to increased generation of free radicals and the occurrence of oxidative stress. High levels of free radicals lead to oxidation of biomolecules resulting in tissue damage, cell death, and delayed burn wound healing [18]. Compounds containing antioxidants can counteract the toxic implications of free radicals and deactivate them. Therefore, the application of antioxidants during the healing process has been the subject of numerous studies [19]. Sahib and colleagues showed that antioxidants reduce the mortality rate of burn patients and play an important role in wound healing. The use of antioxidants and trace elements significantly increased the speed of recovery, prevented complications, and reduced the mortality rate, which positively affected the quality of life, especially considering that most burn patients were disturbed by anxiety [19]. Recently, the influence of natural antioxidants on wound healing has been investigated, and it has been shown that some of them can cause an increase in tissue repair [20,21]. Popularly known as meadowsweet, *Filipendula ulmaria* (FU) is traditionally used in many European and Asian countries. Thanks to its high phenol content, it exhibits antioxidative, anti-inflammatory, and analgesic effects. Knowing this, the use of FU extract in the treatment of post-thermal injuries can lead to multiple benefits [22].

This study aimed to evaluate the impact of the thermal skin injury on the indicators of hippocampal function with the final behavioral outcome. In addition, we intended to estimate the effects of HBO and the antioxidant supplementation with FU extract, individually, in the treatment of anxiety-like behavior that coincides with thermal skin injury, and to compare them to the parameters obtained following their simultaneous administration.

## 2. Results

The applied protocols involving the induction of thermal skin injury resulted in significant alterations of parameters for the estimation of anxiety levels in the behavioral testing, while the treatments with HBO and FU extract on intact animals were ineffective, and therefore were not further commented on considering individual parameters for behavioral alterations, as well as for tissue samples analyses.

**Figure 1:**
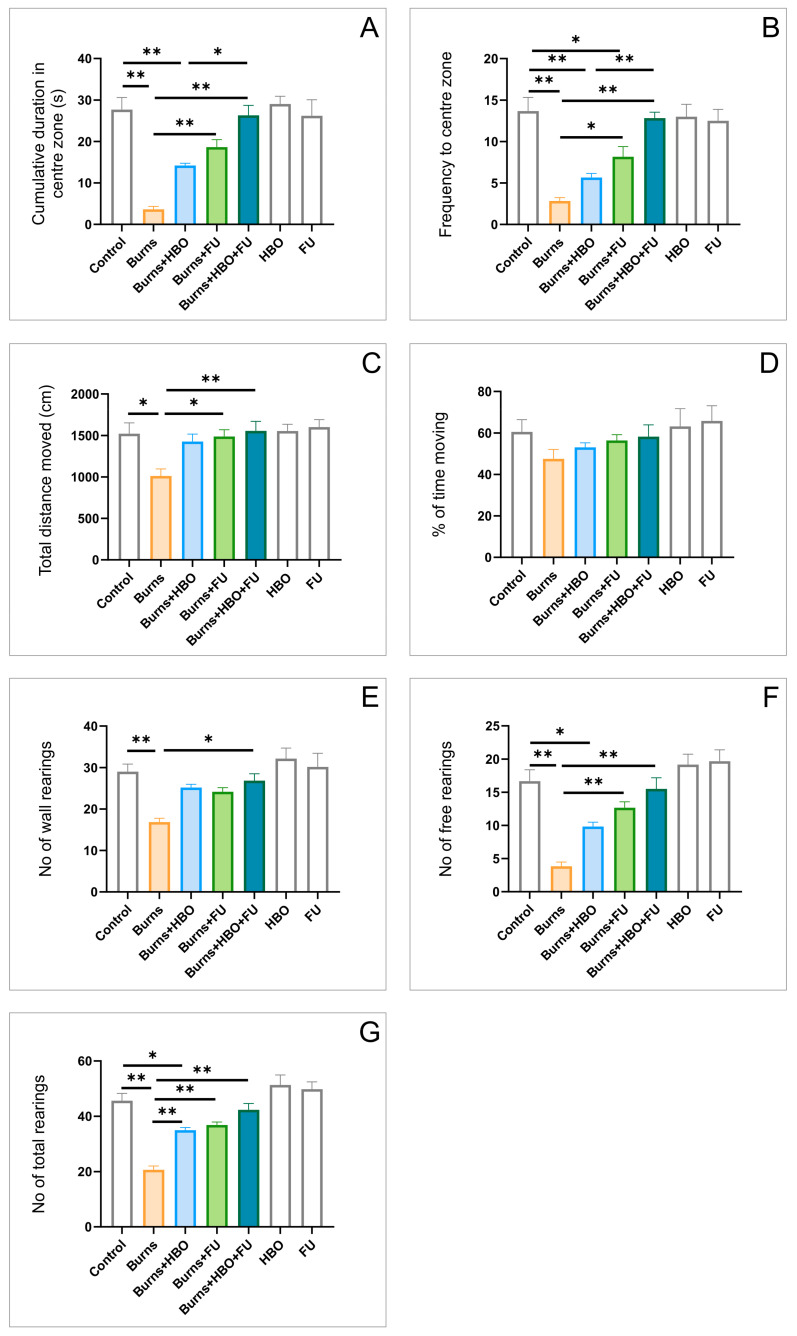
Anxiety level estimation in the open field test (seven equal groups, *n* = 42). (**A**) CDCZ; (**B**) FCZ; (**C**) TDM; (**D**) % TM; (**E**) WR; (**F**) FR; and (**G**) TR. The values are presented as mean ± SEM, (* *p* < 0.05, ** *p* < 0.01).

A total of 8 days after conducting a thermal skin injury, the direct indicators of anxiety obtained in the open field (OF) test, the cumulative duration in center zone (CDCZ, Figure 1A, F = 16.397, df = 6), and the frequency to center zone (FCZ, Figure 1B, F = 13.912) had significant correlation with the anxiogenic response (*p* < 0.01). However, the protocols with FU extract administration alone, and in combination with HBO, significantly enhanced the values obtained in the burns group (*p* < 0.01), although the values obtained with individual FU extract protocol remained significantly below the control values for FCZ (*p* < 0.05). Furthermore, the HBO treatment alone was not sufficient to reverse the burns-induced decline in those parameters. The parameters that count for total motor activity, the total distance moved (TDM, Figure 1C, F = 4.287), and the percentage of time moving (%TM, Figure 1D, F = 1.183) were less affected. With no significant changes for %TM, thermal skin injury again significantly reduced TDM (*p* < 0.05), which was successfully attenuated in the FU extract and combined groups (*p* < 0.05 and *p* < 0.01, respectively), but not in the HBO group. The exploratory activity in OF test was estimated by means of the number of wall (Figure 1E), free (Figure 1F), and total rearings (Figure 1G). The number of wall rearings (WR) was significantly less altered (F = 6.918) when compared to free rearings (FR, F = 17.373), and total rearings (TR, F = 21.769). In all three estimated parameters, the exploratory activity was significantly lowered in the burns group (*p* < 0.01), and successfully increased in the combined group (*p* < 0.01), while the treatment with FU extract resembled the exploratory activity more efficiently than HBO protocol (significantly only for total rearings, *p* < 0.01).

**Figure 2:**
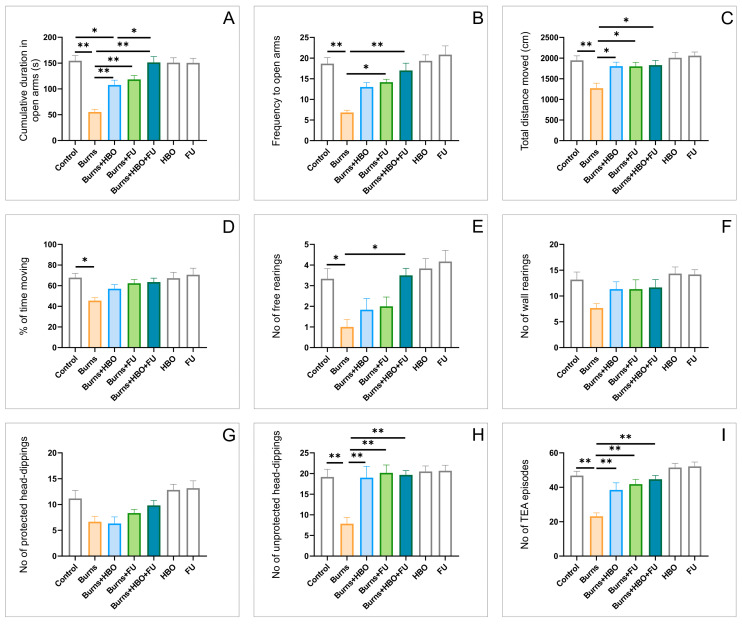
Anxiety level estimation in the elevated plus-maze test (seven equal groups, *n* = 42). (**A**) CDOA; (**B**) FOA; (**C**) TDM; (**D**) % TM; (**E**) FR; (**F**) WR; (**G**) P-HD; (**H**) U-HD; and (**I**) TEA. The values are presented as mean ± SEM, (* *p* < 0.05, ** *p* < 0.01).

Similar observations were made in the elevated plus-maze (EPM) test (Figure 2). Again, the direct parameters of anxiety, the cumulative duration in open arms (CDOA), and frequency to open arms (FOA, Figure 2A,B), were significantly affected by the applied protocols (F = 16.653 and 11.716, respectively). Both parameters declined significantly (*p* < 0.01) in the burns group, which was significantly (*p* < 0.01) reversed in the combined group. Individual administration of FU extract significantly increased the values of CDOA (*p* < 0.01) and FOA (*p* < 0.05), while HBO treatment was successful only for CDOA (*p* < 0.01). Like the OF test, TDM and %TM were significantly lowered by peripheral thermal injury (Figure 2C, F = 5.677 and 3.626, *p* < 0.01 and 0.05, respectively), and this was successfully reversed by all applied protocols (*p* < 0.05, for TDM), achieving the control values for %TM. The exploratory activity, analyzed using the free rearings, was significantly altered by the applied protocols (Figure 2E, F = 2.847), but not for the wall rearings (Figure 2F, F = 6.581), where the burns resulted in a significant decline in free rearings (*p* < 0.05), and this was reversed in the combined group (*p* < 0.05). The other patterns of exploratory activity in the EPM test, and the number of protected (P-HD) and unprotected head-dippings (U-HD) (Figure 2G,H, respectively), showed a similar response to the applied protocols. With no significant alterations in the number of P-HD (F = 6.779), U-HD (F = 5.534) significantly declined following thermal skin injury (*p* < 0.01), which was significantly altered by all three protocols for the treatment of the burns (*p* < 0.01). This also affected the number of the total exploratory activity (TEA) episodes (Figure 2I, F = 13.049), resulting in the same responses.

**Figure 3:**
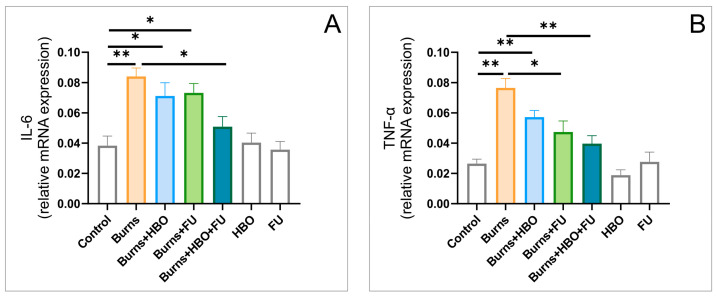
Cytokines alterations in the hippocampal tissue (relative gene expression, seven equal groups, *n* = 42). (**A**) IL-6; (**B**) TNF-α. The values are presented as mean ± SEM, (* *p* < 0.05, ** *p* < 0.01).

The analyses of the relative cytokines expression in the hippocampal tissue showed that the expression of both IL-6 and TNF-α was significantly altered (Figure 3A,B, F = 8.956 and 14.150, respectively). The relative expression of pro-inflammatory cytokines was significantly enhanced in the burns group (*p* < 0.01), and sufficiently attenuated by FU extract administration alone (for TNF-α, *p* < 0.05), as well as in combination with HBO treatment for both cytokines.

**Figure 4:**
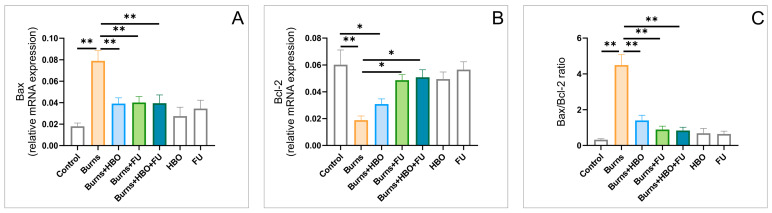
Apoptotic markers alterations in the hippocampal tissue (relative gene expression, seven equal groups, *n* = 42). (**A**) Bax; (**B**) Bcl-2; (**C**) Bax/Bcl-2 ratio. The values are presented as mean ± SEM, (* *p* < 0.05, ** *p* < 0.01).

The apoptotic activity, estimated by the relative expression of pro- and anti-apoptotic markers in the hippocampal tissue, Bax and Bcl-2 (Figure 4A,B), following the described experimental design, was significantly affected (F = 7.356 and 5.992, respectively). While the Bax relative gene expression was significantly augmented after the thermal skin injury (*p* < 0.01), Bcl-2 expression was diminished (*p* < 0.01) 8 days after the peripheral injury in the burns group. All protocols applied for treatment for initial lesions successfully lowered the burns-induced increase in the Bax expression (*p* < 0.01), but only the protocols with antioxidant supplementation with FU extract resulted in significant recovery of the Bcl-2 expression (*p* < 0.05). Not surprisingly, the apoptotic index (Bax/Bcl-2 ratio, Figure 4C) followed the same algorithm as observed for the Bax relative gene expression (F = 24.192), with the significantly increased ratio in the burns group (*p* < 0.01), which was reversed by the applied protocols (*p* < 0.01).

**Figure 5:**
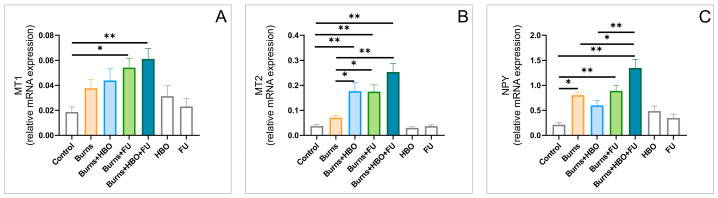
Alterations of melatonin receptors and NPY in the hippocampal tissue (relative gene expression, seven equal groups, *n* = 42). (**A**) MT1; (**B**) MT2; (**C**) NPY. The values are presented as mean ± SEM, (* *p* < 0.05, ** *p* < 0.01).

The analyses of the relative gene expression of melatonin receptors (MT1 and MT2, Figure 5A,B) in the hippocampal tissue showed significant alterations following the applied protocols (F = 4.460 and 16.842, respectively). With no significant increase 8 days after the thermal skin injury itself, the applied protocols with FU extract (BURNS+FU and BURNS+HBO+FU) significantly increased MT1R expression when compared to the control values (*p* < 0.05 and 0.01, respectively). MT2R expression was significantly increased by the protocols applied after the burns when compared to the intact animals (*p* < 0.01), but even more when compared to the burns group (*p* < 0.05 for individual, and 0.01 for the combined administration). As shown in Figure 5C, the significant alterations (F = 11.818) in neuropeptide Y (NPY) hippocampal relative gene expression were manifested as a significant increase in the burns group (*p* < 0.05), as well as in BURNS+FU and BURNS+HBO+FU groups (*p* < 0.01), when compared to the control values. Also, the combined group showed a significant increase in NPY expression when compared to the burns group (*p* < 0.05).

**Figure 6:**
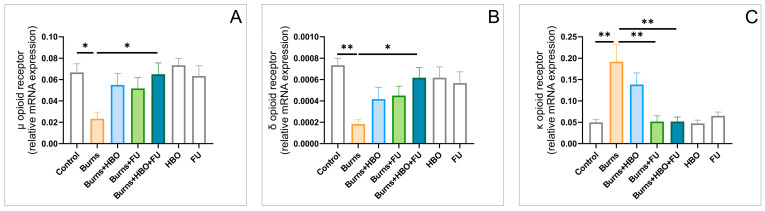
Alterations of opioid receptors in the hippocampal tissue (relative gene expression, seven equal groups, *n* = 42). (**A**) µ receptors; (**B**) δ receptors; (**C**) κ receptors. The values are presented as mean ± SEM, (* *p* < 0.05, ** *p* < 0.01).

The relative gene expression of the opioid receptors µ, δ, and κ (Figure 6A–C, respectively) in the hippocampal tissue was significantly influenced in this experimental design (F = 3.373, 4.071, and 8.129, respectively). While the expression of µ and δ receptors was up-regulated after the thermal skin injury (*p* < 0.05 for µ, and 0.01 for δ receptors) and restored in the combined group (*p* < 0.05), the opposite response was observed for κ receptors hippocampal expression. Namely, a significant increase in κ receptors was observed in the burns group (*p* < 0.01), when compared to the control, and this was significantly attenuated by both protocols that assumed FU extract administration (*p* < 0.01).

**Figure 7:**
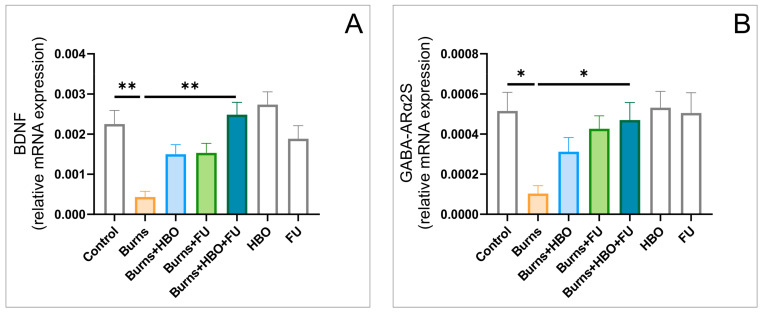
Alterations of BDNF (**A**) and GABA-ARα2S (**B**) in the hippocampal tissue (relative gene expression, seven equal groups, *n* = 42). The values are presented as mean ± SEM, (* *p* < 0.05, ** *p* < 0.01).

The hippocampal tissue samples analyses revealed that the relative gene expression for the brain-derived neurotrophic factor (BDNF) and GABAA receptor alpha-2 subunit (GABA-ARα2S, Figure 7A,B) was significantly changed by the described procedures (F = 7.576 and 3.805) in almost the same manner. The thermal skin injury resulted in a significant decline for both BDNF (*p* < 0.01) and GABA-ARα2S (*p* < 0.05) gene expression, which was successfully recovered in the combined group (*p* < 0.01 for BDNF and 0.05 for GABA-ARα2S).

**Figure 8:**
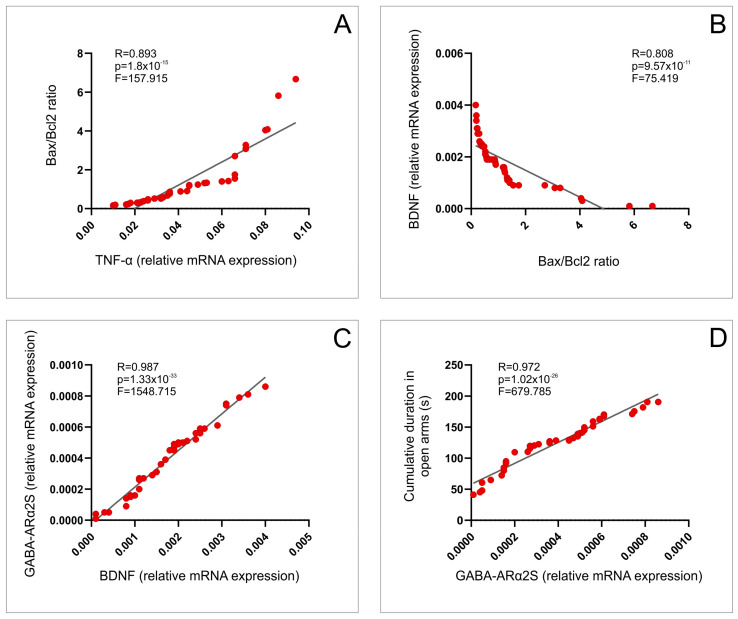
Relationship between TNF-α and Bax/Bcl-2 ratio (**A**); Bax/Bcl-2 ratio and BDNF (**B**); BDNF and GABA-ARα2S (**C**); (**D**) GABA-ARα2S (relative gene expression) and CDOA during EPM test.

The linear regression analysis revealed that the increase in TNF-α relative gene expression significantly (positively) correlated with the increase in apoptotic ratio in the rat hippocampus (Figure 8A, *p* < 0.01), which, in turn, negatively correlated to the relative BDNF gene expression (Figure 8B, *p* < 0.01). Furthermore, the decline in BDNF expression strongly correlated to the down-regulation of GABA-ARα2S (Figure 8C, *p* < 0.01), and finally, to the anxiogenic-like response expressed by the lowered CDOA (Figure 8D, *p* < 0.01).

**Figure 9:**
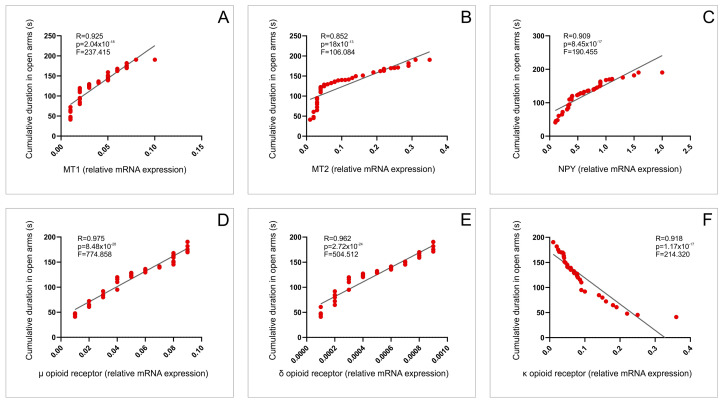
Relationship between the hippocampal expression of receptors involved in pain control and anxiety level: MT1 (**A**), MT2 (**B**), NPY (**C**), µ opioid (**D**), δ opioid (**E**), κ opioid (**F**) receptors relative gene expression and CDOA during EPM test.

The same analysis also showed that the hippocampal relative gene expression of MT1 and MT2 receptors (Figure 9A,B), as well as NPY expression (Figure 9C), positively correlated with the CDOA (*p* < 0.01). At the same time, the positive correlation to this anxiolytic marker was confirmed for µ and δ opioid receptors (Figure 9D,E, *p* < 0.01), while there was a negative correlation for the relationship between κ receptors gene expression and CDOA (Figure 9F, *p* < 0.01).

## 3. Discussion

Since it has been confirmed that the reaction to hyperthermic skin injury is considered a chronic intermittent stress response [23], it was not surprising that the results obtained in this study showed significant behavioral alterations manifested as a clear anxiogenic response to post-burn pain. Indeed, the observed algorithm of pain behavior in this investigation was expressed through the rise in both direct and indirect anxiety indicators in OF and EPM tests (Figure 1 and Figure 2). This outcome of the skin thermal wound is in line with the previous clinical evidence [8]. Nevertheless, due to the lack of standardization in the rat thermal burn model [24], the problems with the quantification of the observed behavioral alterations are persisting, and this allows for the comparison with the other studies. As previously mentioned, the performed treatments with HBO and antioxidant supplementation significantly affected the parameters for anxiety level estimation. It seems that the applied protocols attenuated the post-burn, pain-induced, and anxiogenic-like behavior. Of course, the behavioral benefits of the performed treatments could be attributed to their previously described diminishing impact on primary skin lesions [25], but in this investigation, we focused on the mechanisms that simultaneously occur in the hippocampus, since it has been confirmed for the regulation of anxiety levels [26]. Although there are no literature data for this specific source of antioxidants (*Filipendula ulmaria*), this methodological approach was strongly supported by the findings that interventions involving the restoration of oxidative equilibrium by applying other antioxidant supplementation (*Lavandula angustifolia*) could simultaneously result in an antinociceptive and anxiolytic-like outcome [27]. Interestingly, HBO treatment has also shown its benefits in preconditioning post-traumatic stress disorder in rats [28].

The analysis of hippocampal tissue, as shown in Figure 3, showed that 7 days after a thermal injury, the relative expression of estimated pro-inflammatory cytokines (IL-6 and TNFα) was significantly above the control values. This observation is in accordance with previous findings [29] that confirm an almost simultaneous increase in pro-inflammatory cytokines in peripheral blood immediately after a peripheral thermal injury. Interestingly, this pro-inflammatory reaction was also registered in the brain tissue, and this occurrence was at least partially attributed to the disturbance of the blood–brain barrier [30]. On the other hand, this prolonged elevation in the pro-inflammatory cytokines profile was successfully attenuated by the applied plant extract and HBO, reaching the control values in the combined group. Our results correspond to the previously obtained reduction in the inflammatory response in critically ill burn patients achieved with multivitamins and infusions of trace elements [31], as well as with the reported anti-inflammatory action of FU extract in rats [22]. This neuroinflammatory pattern that occurred along with the post-burn, pain-induced anxiety response was concomitant with the pro-apoptotic action, probably of the same origin. It was manifested through the increase in the relative expression of the pro-apoptotic (Bax) and a decline in the anti-apoptotic (Bcl-2) markers. This finding is in line with the previously described brain injury following peripheral burn injury that was manifested in neurons and microglia [5]. Again, all protocols applied to minimize the thermal wound and improve healing simultaneously and successfully reversed the pro-apoptotic alterations in the hippocampal tissue in this study (Figure 4). The benefits of antioxidants-supported therapy in the treatment of brain injury of different origins had been previously reported [32], but with a recommendation that different antioxidant classes [33], including FU extract, should be used to diminish apoptotic events in hippocampal tissue. On the other hand, HBO treatment, as the other therapeutic entity employed in this study, showed beneficial effects in preventing apoptotic events following traumatic spinal cord [34] and brain injury [35], although via different pathways.

The analysis of the relative gene expression in the rat hippocampus samples also revealed a significant decline in BDNF. Similar observations were previously reported for the chronic stress-induced reduction in BDNF mRNA expression in the rat hippocampus [36], while this occurrence was also confirmed for the other neurotrophins [37]. However, the benefits of therapeutic protocols with antioxidant supplementation and HBO were also confirmed by the increase in hippocampal BDNF relative expression (Figure 7). This is in accordance with the beneficial effects of antioxidant supplementation with matrine reported for neurobehavioral alterations in a mouse model of burn injury [38], as well as with our previous reports that showed the increase in BDNF content following prolonged administration of FU extract in rat hippocampus and prefrontal cortex [39,40]. On the other hand, the neuroprotective role of HBO therapy was previously reported in in vitro on spiral ganglion neurons [41] and in a clinical trial with delayed encephalopathy [42], but with the opposite effect on BDNF in dorsal horns of the spinal cord [17]. 

The chronic pain-induced alterations in hippocampal tissue following thermal injury were also accompanied by the decreased relative expression of GABA-AR (Figure 7). This is in line with the finding that injury-induced chronic pain is accompanied by the general down-regulation of most GABA-AR channel subunits in rats’ peripheral sensory ganglia [43]. Again, the applied protocols reversed the diminishing of GABA-AR after 7 days. The most prominent recovery of the hippocampal GABAergic system was observed in the combined group, supporting the previous findings that confirm the up-regulation of GABA-AR in the rat hippocampus following the intake of antioxidants [40]. Our results also correspond to the reported GABA-A (but not GABA-B) receptors antinociceptive effect in mice spinal cord that was significantly augmented with HBO therapy [44].

As shown in Figure 8, it seems that the initial pro-inflammatory and pro-apoptotic impact of a thermal injury may contribute to the lowering of hippocampal BDNF by affecting neurons after peripheral burns [5]. This may be attributed to the cut down of GABA-AR expression [45], and consequent anxiogenic response, as previously described by Stajic and coworkers [46]. 

**Figure 10:**
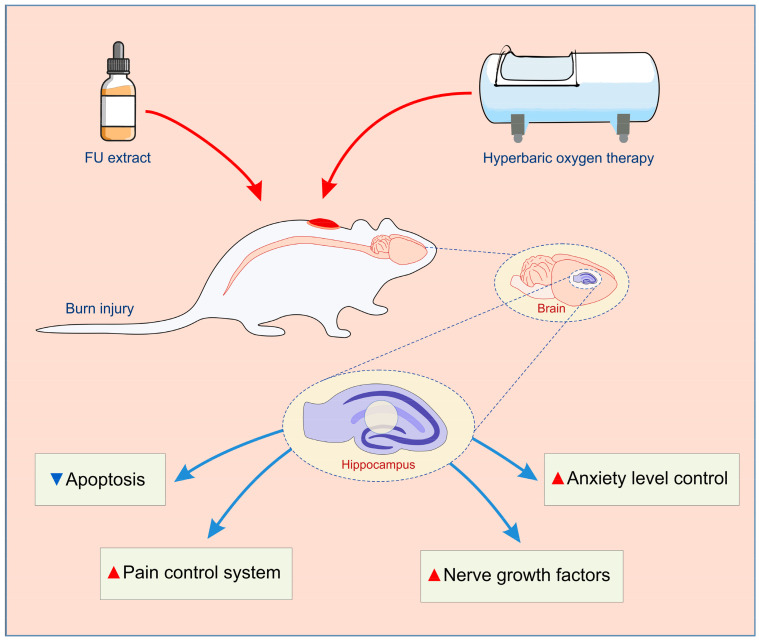
The multiple pathways for anxiolytic response induced by HBO and antioxidant supplementation with FU extract following the thermal skin injury (blue triangle—down-regulation; red triangle—up-regulation).

To allow better insight into the potential roles of the specific receptors involved in pain control, we also estimated the impact of the skin burn wounds, as well as the described therapeutic protocols, on their relative gene expression in the rat hippocampus. Thus, we observed that both MT1 and MT2 receptor gene expression were insignificantly up-regulated 7 days after the thermal injury. On the other hand, the applied protocols resulted in a significant increase in those hypoalgesic receptors’ hippocampal expressions, with the most prominent effect in the combined group. Indeed, when analyzing the impact of melatonin receptors, the principal fact that melatonin itself has antioxidant properties should be taken into account [47]. The increased hippocampal relative mRNA expression of MT1 and MT2 receptors observed in this study following HBO treatment is in line with the previously reported up-regulation of melatonin receptors in the spinal cord [17]. Similar observations were obtained for NPY hippocampal expression, in which this hypoalgesic peptide expression was reactively enhanced following skin burns, but even more increased after the described therapeutic protocols. Namely, the increased relative expression of NPY in rat hippocampus following FU extract intake is in accordance with the similar response observed in mice hypothalamus after ginseng administration [48]. The presented enhancement in the hippocampal NPY expression following HBO treatment is in line with the previously described NPY up-regulation achieved in mice exposed to HBO arcuate nucleus [49]. The significant alterations for opioid receptors relative gene expressions in the hippocampus were also reported in the study that estimated the neuropathic pain, although in the different portions of CNS [50]. Nevertheless, the changes in the opioid receptors hippocampal expression, as the result of the protocols performed in this study, did not follow the same algorithm, since the diminished burns-induced expression of µ and δ receptors, in combination with the significant enhance of κ receptors expression, were successfully reversed by the applied protocols. Our findings considering the hyperbaric protocol effect are in accordance with the response in the sciatic nerve-crushed rats by means of the enhanced opioid receptors expression [51], which was more pronounced in the different experimental model (burn-induced neuropathic pain) [17]. Still, it should be noticed that the simultaneous increase in µ and κ receptors in different brain and peripheral regions following HBO protocols, as previously described [17], was not in line with the observed decline in κ receptors expression in this study. Although the studies were performed in the same species, it seems that some specific tissue differences may be attributed to the reported differences in the opioid system response. Thus, it seems that the alterations in pain control receptors’ hippocampal expression were also sensitive to skin burn injury, as well as to the applied protocols. Their specific changes may at least quantitatively contribute to the observed behavioral alterations (Figure 9), according to the previously presented relationship between the levels of nociception and anxiety [52] in genetically modified animals, which may involve GABAergic receptors [53], Figure 10. 

## 4. Materials and Methods

### 4.1. Housing

In this investigation, we used male *Wistar albino* rats (200–250 g), eight to ten weeks old, purchased from the Military Medical Academy, Belgrade, Serbia. The housing was organized in two plexiglass cages per group of animals with the free continual access to water and food, under standard light/dark cycles, and other environmental conditions. After one-week habituation period, the animals (42 in total) were randomly divided into seven equal groups: CONTROL, BURNS, BURNS+FU, BURNS+HBO, BURNS+FU+HBO, HBO, and FU group. 

### 4.2. Treatment

#### 4.2.1. Thermal Skin Injury Experimental Model

A total of 24 animals (BURNS, BURNS+HBO, BURNS+FU, and BURNS+HBO+FU) were weighed and anesthetized with the combination of ketamine and xylazine (10 and 5 mg/kg, i.p., respectively). Antisepsis was achieved by local application of 1% polyvinylpyrrolidone iodine before trichotomy of a back area measuring approximately 3 cm^2^ while the thermal skin injuries were induced using the electrically driven solid aluminum bar (10 mm in diameter), to achieve the constant temperature of 75 °C for 15 s on the preferred (dorsal proximal thoracic) region [26,54], following the exact previously described procedure taking into account clinical and histological characteristics [26].

#### 4.2.2. Hyperbaric Oxygen Treatment (HBO) and Antioxidant Supplementation with *Filipendula ulmaria* (FU) Extract

A hyperbaric chamber for rats (HYB-C 300) was used for HBO protocol for seven consecutive days (100% O_2_ at 2.5 ATA for 60 min, 24 h after the induction of burns), as previously described [55], following the predefined schedule (1:00–8:00 PM, three animals in a chamber per session) in all groups that included this therapeutic approach.

Antioxidant supplementation was performed, as previously described in detail [39], with *Filipendula ulmaria* extract [22], for seven consecutive days (also starting 24 h following the induction of burns), to achieve the final daily dose of 100 mg/kg b.w., in all groups that included this therapeutic approach. The final concentration of FU extract was achieved by free access to drinking water based on the total water intake on the previous day.

### 4.3. Anxiety Level Estimation

Behavioral testing was performed following the completion of the seven-day therapeutic protocols. The animals were accommodated in the testing area for one hour (app. at 8 AM) and tested using two specific tests: an open field (OF), followed by an elevated plus-maze (EPM) test, which lasted for five minutes (intertrial interval of 15 min), following the procedures to eliminate potentially interfering elements that might affect the test. Video recordings were analyzed with Ethovision software XT 13.0.1220 (Noldus Information Technology, Wageningen, The Netherlands). In that manner [56], we were able to obtain the following parameters from the OF test:

CDCZ (in s), FCZ, TDM (in cm), %TM, the number of WR, FR, and TR. This methodology also allowed to obtain the following parameters from the EPM test;

CDOA (in s), FOA, TDM (in cm), %TM, the number of WR and FR, the number of P-HD and U-HD, and the number of TEA episodes.

### 4.4. Hippocampal RNA Isolation and Real-Time PCR Analysis

After the sacrifice hippocampi were isolated, snap-frozen, and then stored at −80 °C. Total RNA was extracted using PureZOL reagent (Bio-Rad, Hercules, CA, USA). Reverse transcription was carried out using iScript Reverse Transcription Mastermix (Bio-Rad, Hercules, CA, USA). Quantitative RT-PCR was performed using SsoAdvanced Universal SYBR Green Supermix (Bio-Rad, Hercules, CA, USA). mRNA-specific primers (Supplementary File Table S1) for IL-6, TNF-α, Bax, Bcl-2, μ opioid receptor (MOR), δ opioid receptor (DOR), κ opioid receptor (KOR), melatonin receptors (MT1, MT2), neuropeptide Y (NPY), BDNF, GABA-ARα2S, were used, including β-actin as housekeeping gene (Invitrogen, Waltham, MA, USA). RT-PCR reactions were carried out using the Bio-Rad CFX96 (Bio-Rad, Hercules, CA, USA). Relative gene expression was analyzed as previously described by Livak and Schmittgen [57].

For all analyses, the investigators were blind to previously performed protocols (treatment groups), and for behavioral parameters all data were obtained using the program incorporated in the software.

All research procedures were carried out in accordance with the European Directive for the welfare of laboratory animals No 86/609/EEC, the principles of Good Laboratory Practice, and in accordance with the ARRIVE guidelines. The study was approved by the Ethical Committee of the Faculty of Medical Sciences, University of Kragujevac, Serbia.

### 4.5. Statistical Analysis

Statistical analysis was performed using IBM software package SPSS 20.0. The results are expressed as the means ± standard errors of the mean (SEM). The parameters were initially submitted to Levene’s test for homogeneity of variance and to Shapiro–Wilk test of normality. One-way ANOVA, followed by Bonferroni test, was used for comparisons between the groups. The significance was determined at *p* < 0.05 for all tests. Simple linear regression and Pearson’s coefficient of correlation were used to analyze the relationships between the obtained parameters.

## 5. Conclusions

Taken altogether, since the thermal skin injury-induced chronic pain, along with second-morbidities, and also produces emotional reactions, such as anxiety, it can be assumed that future investigations of potential therapeutic protocols to treat skin wounds should include the estimation of their action in brain regions involved in emotional regulations. Also, it seems that both antioxidant supplementation and HBO may beneficially contribute to achieving optimal mental abilities earlier, and therefore improve overall recovery. Finally, the results of this study bring some confirmations that adjuvant strategies, such as HBO and antioxidant supplementation, may be simultaneously applied in the treatment of the anxiety-like behavior that occurs with thermal skin injury.

## Data Availability

Data are available upon request.

## Supplementary Materials

The following supporting information can be downloaded at: https://www.mdpi.com/article/10.3390/ijms25053033/s1.
