# Supplementary material for: Complex Hippocampal Response to Thermal Skin Injury and Protocols with Hyperbaric Oxygen Therapy and Filipendula ulmaria Extract in Rats
Source: Int J Mol Sci. 2024 Mar 6;25(5):3033. doi: 10.3390/ijms25053033 (PMC10932411; doi:10.3390/ijms25053033)
Supplement: Supplementary file 1 [file ijms-25-03033-s001.zip › ijms-2873963-supplementary.pdf]

Supplement Table S1. RT-PCR primers used in this study.

	**Forward**	**Reverse**
β-actin	AAGATCCTGACCGAGCGTGG	CAGCACTGTGTTGGCATAGAGG
Bcl-2	TGTGGATGACTGACTACCTGAACC	CAGCCAGGAGAAATCAAACAGAGG
Bax	CGGCGAATTGGAGATGAACTGG	CTAGCAAAGTAGAAGAGGGCAACC
GABA-ARα2S	TTA CAG TCC AAG CCG AAT GTC CC	ACT TCT GAG GTT GTG TAA GCG TAG C
BDNF	AGCTGAGCGTGTGTGACAGT	ACCCATGGGATTACACTTGG
IL-6	TCCTACCCCAACTTCCAATGCTC	TTGGATGGTCTTGGTCCTTAGCC
TNF-α	AAATGGGCTCCCTCTCATCAGTTC	TCTGCTTGGTGGTTTGCTACGAC
MOR	CATATTCACCCTCTGCAC	TTACAGGCAGACCGATG
DOR	TTACAGGCAGACCGATG	ATGTTTGGAATCGTCCGGTACA
KOR	TCTAGCTATTACTTCTGCATTG	TGTGTTTCTAACTCTGTTTGT
MT1	GCCACAGTCTCAAGTATGATAGG	GGTGACAAAGTTCCTGAAGTC
MT2	CCTCTACATCAGCCTCATCTGGCT	CTGCGAACATGGTTAGGAAACTGC
NPY	TCTGCCTGTCCCACCAATG	CAACGACAACAAGGGAAATGG
